# Cryoconite as a temporary sink for anthropogenic species stored in glaciers

**DOI:** 10.1038/s41598-017-10220-5

**Published:** 2017-08-29

**Authors:** Giovanni Baccolo, Biagio Di Mauro, Dario Massabò, Massimiliano Clemenza, Massimiliano Nastasi, Barbara Delmonte, Michele Prata, Paolo Prati, Ezio Previtali, Valter Maggi

**Affiliations:** 1 Department of Environmental Sciences, University of Milano-Bicocca, P.zza della Scienza 1, 20126 Milano, Italy; 2 INFN, section of Milano-Bicocca, P.zza della Scienza 3, 20126 Milano, Italy; 3 Department of Physics, University of Genova, Via Dodecaneso 33, 16146 Genova, Italy; 4 INFN, section of Genova, Via Dodecaneso 33, 16146 Genova, Italy; 5 University of Milano-Bicocca, Physics Department, P.zza della Scienza 3, 20126 Milano, Italy; 6 LENA, University of Pavia, Via G. Aselli 41, 27100 Pavia, Italy

## Abstract

Cryoconite, the typical sediment found on the surface of glaciers, is mainly known in relation to its role in glacial microbiology and in altering the glacier albedo. But if these aspects are relatively well addressed, the same cannot be said about the geochemical properties of cryoconite and the possible interactions with glacial and peri-glacial environment. Current glacier retreat is responsible for the secondary emission of species deposited in high-altitude regions in the last decades. The role played by cryoconite in relation to such novel geochemical fluxes is largely unknown. Few and scarce observations suggest that it could interact with these processes, accumulating specific substances, but why, how and to what extent remain open questions. Through a multi-disciplinary approach we tried to shed lights. Results reveal that the peculiar composition of cryoconite is responsible for an extreme accumulation capability of this sediment, in particular for some, specific, anthropogenic substances.

## Introduction

Cryoconite is a dark incoherent-granular sediment found on the ablation surface of glaciers. Its name, from the ancient Greek κρύον (cold/ice) and κόνις (dust), was coined by the Swedish explorer Adolf Erik Nordenskiöld during its exploration of inner Greenland in 1882–83^1^. Few years later its presence was reported also in the Alps^2^ and successively on glaciers around the world. Initially it was considered merely as a glaciological curiosity, but now cryoconite is drawing attention from many fields of Earth and Life sciences^3, 4^. Because of its dark color a first immediate impact concerns the radiative and optical properties of glaciers where it is accumulated^5, 6^. This is true both considering single glaciers or continental ice sheets found in polar regions^5, 7^. Another essential aspect regards its importance as a hotspot for microbial life and its link with global carbon cycle^8–10^.

But what is exactly cryoconite? Its composition is complex. Two fractions are distinguished: a mineral fraction and an organic one. The mineral fraction is dominant, accounting for 85–95% of total mass, while the remnant fraction is composed by organic matter^3^ (OM). Mineral dust found on the glacier surface acts as a substrate for microbial organisms which start growing and building structures and bio-films which consolidate and darken the resulting sediment^5, 11^.

The relations between the composition of cryoconite, the glacial environment and atmospheric depositional fluxes are aspects which have been only marginally explored. Glaciers and snow are known to record both natural and anthropogenic atmospheric emissions^12^. Current glacial melting poses critical issues in this context, since pollutants accumulated and entrapped within ice bodies during last decades are now being re-mobilized^13–15^. Given its physical and chemical properties cryoconite could play a role in this process. The contemporary presence of fine mineral material and organic matter could enhance the adsorption and accumulation in cryoconite of both organic and inorganic substances. The few available data suggest that something similar is actually taking place^16, 17^. This sediment could thus play a role in determining the fate of pollutants stored in glaciers and released in the environment as a consequence of glacier retreat.

**Figure 1:**
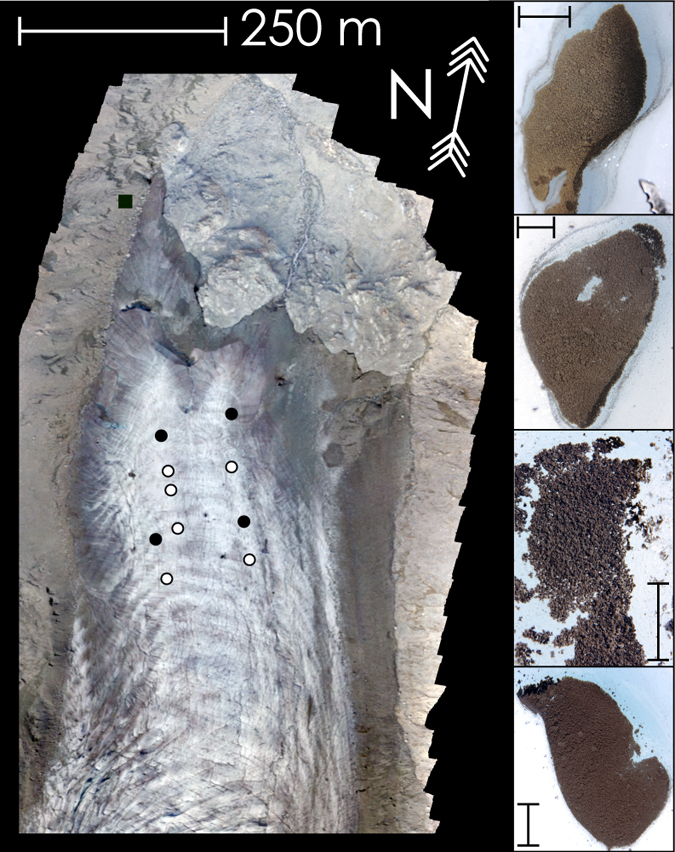
The terminal part of the Morteratsch glacier from a photogrammetric reconstruction (September 2016). Cryoconite samples collection sites are indicated by dots (black 2015; white 2016), the black square is used for moraine sediment collection site). On the right some cryoconite holes sampled to the aim of this work; 5 cm bar is reported.

To better comprehend the dynamics which control these processes, we analyzed the composition of cryoconite collected from a glacier in the Swiss Alps (Fig. 1). To obtain a comprehensive picture, attention was paid to radioactivity, elemental geochemistry and carbonaceous content, determined respectively through γ- spectroscopy, instrumental neutron activation analysis (INAA) and thermo-optical analysis.

The Alps are set in the middle of Europe. Being surrounded by some of the most industrialized and inhabited regions of the planet. Their position is extremely sensitive to atmospheric pollution^12, 18^, including radioactive fallout associated to punctual accidents and to global contamination^19–21^. Such features make Alpine glaciers an ideal terrain to better comprehend the role played by glaciers and cryoconite in determining the fate of many natural and anthropic atmospheric species in glacial environments.

## Results and Discussion

**Figure 2:**
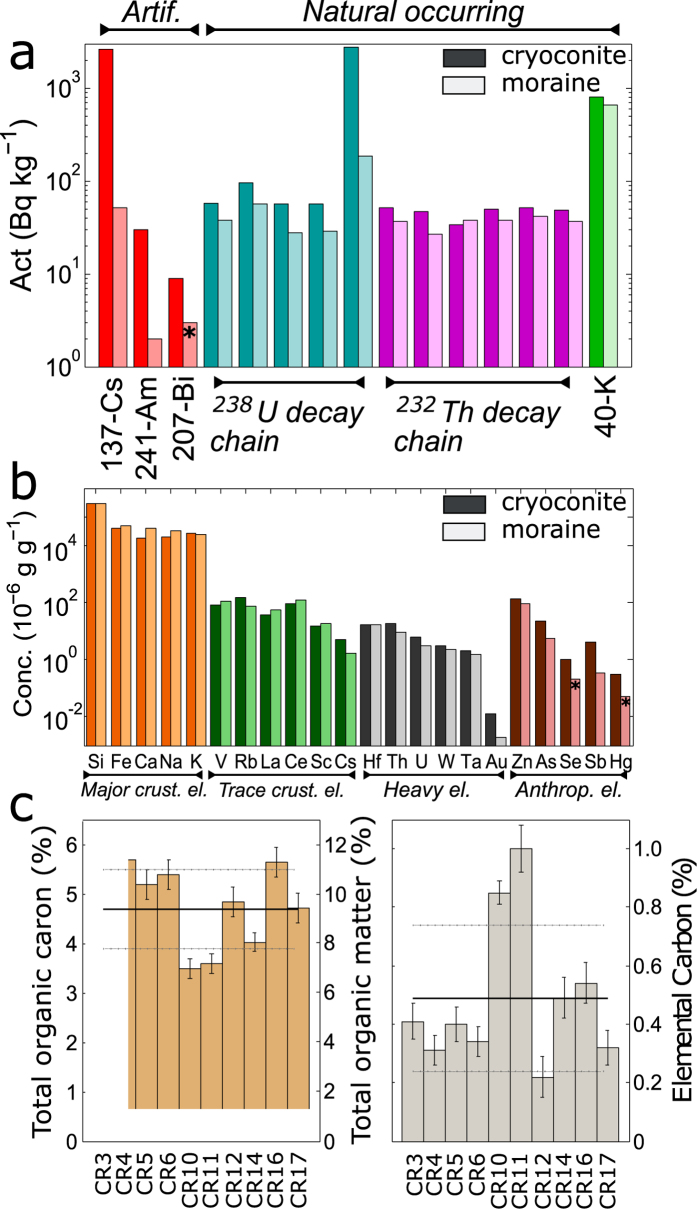
Average data about natural radioactivity (panel a), elemental composition (panel b) and carbonaceous content (panel c). In the first two panels average results about cryoconite and moraine sediments are presented (dark and light colors respectively). Not all nuclide labels are reported for U and Th chains, see Fig. 3 for the complete list. In panel c data related to single sample are shown, lines refer to average and standard deviation values. Asterisks highlight to values below detection limit.

**Figure 3:**
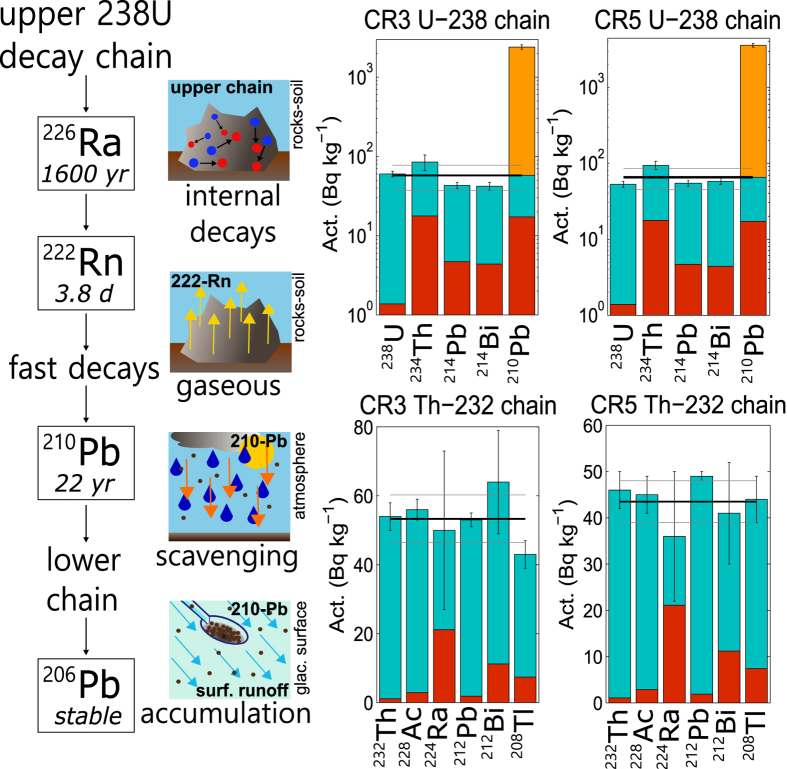
^238^U and ^232^Th decay chains in relation to cryoconite. On the left a scheme presenting the decay chain of ^238^U and the interactions between ^210^Pb and cryoconite. On the right the activity of radionuclides belonging to the ^238^U chain (upper part, logarithmic scale) and to the ^232^Th one (lower part). Data about two cryoconite samples (CR3 and CR5) are shown. Chains are partial and represented from left to right. Detection limits (red bars) and observed activities (blue bars) are presented with relative errors. Black (grey) line(s) refers to mean (st. dev.) activity of the entire decay chains. In the case of Pb-^210^ supported (blue bar) and unsupported (orange bar) fractions are highlighted.

**Figure 4:**
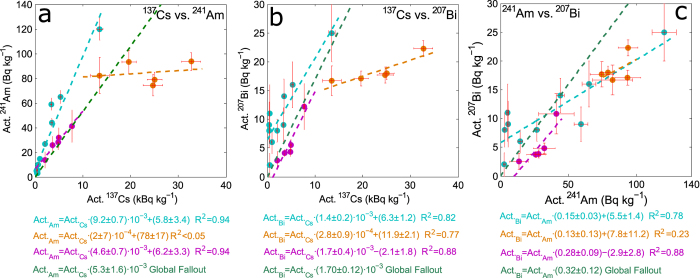
Artificial radionuclides observed in Alpine cryoconite. Our data from the Morteratsch glacier (light blue) are compared to cryoconite collected from Italian^36^ (violet) and Austrian glaciers^16^ (orange). All data are corrected for January 2017. For each pair of nuclides linear regression is applied and the associated equation presented. Green line refers to global fallout reference^29, 37^.

**Figure 5:**
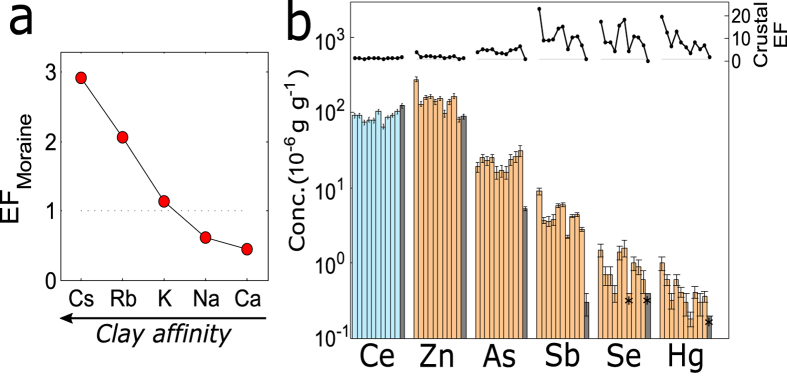
Elements presenting an enrichment in cryoconite with respect to moraine sediments. In panel a the average concentration of cationic elements in cryoconite, normalized with respect to moraine sediment composition, is shown. Elements are ordered following their affinity for clay minerals^42, 43^. In panel b full data about the anthropic elemental group, an additional crustal element (Ce, light blue bars) is included for comparison. For each element data are presented as absolute concentration (bars, lower part) and as enrichment factor with respect to UCC (black curve, upper parts). Moraine sediment data are also presented (grey bars). Asterisks are referred to values below the detection limit.

**Figure 6:**
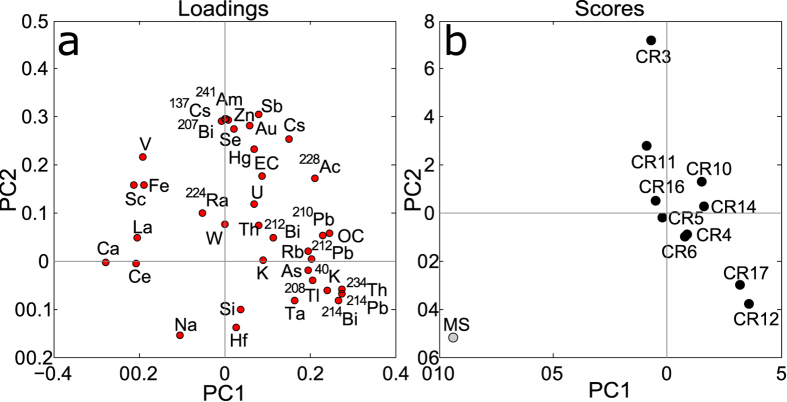
Results about the first two principal components, together they display about 60% of total data variance. In panel a it is shown the influence of the original variables with respect to PCs (loadings plot). Panel b shows the projection of the original samples in the new multivariate space (scores plot, cryoconite samples in black, moraine sediment, MS, in grey).

Full data, including Pearson correlation coefficients are found in the Supplementary Material. In Fig. 2 a wide selection of data is graphically presented, more detailed data are shown in Figs 3, 4 and 5. Principal component analysis main results are presented in Fig. 6.

### Environmental radioactivity

The analysis of γ-spectra revelaed the presence of both natural and artificial radionuclides. Average activities concerning both the categories are shown in Fig. 2a. Natural species belong to ^238^U and ^232^Th decay chains or are primordial radioactive nuclei (^40^K). Using data concerning total U and Th concentrations, determined through INAA, it was also possible to calculate the activity of primordial ^238^U and ^232^Th, partially reconstructing their decay chains (two examples displayed in Fig. 3).

The activity values of natural species are in perfect agreement with common environmental ones, with the exception of ^210^Pb. Its activity reaches an average value of 2.8 *kBq* *kg* ^−1^, with a peak of 4.2 *kBq* *kg* ^−1^, well above what reported in environmental matrices^22^. The biogeochemical cycle of ^210^Pb is complex, being related to the diffusion in the atmosphere of the radioactive gas ^222^Rn. A scheme of its cycle is given in Fig. 3. Both the nuclides are intermediate products along the ^238^U decay chain. The peculiarity of ^222^Rn is its possibility to escape minerals and rocks diffusing into the atmosphere, where it rapidly decays to ^210^Pb^22^. Its presence in the environment is related both to local production due to intrinsic uranium decay (supported ^210^Pb) or to the deposition from the atmosphere in association to precipitations (unsupported or excess ^210^Pb). Comparing the activity of upper and lower chains, it is possible to distinguish the two fractions^23^. In cryoconite the unsupported fraction is on average 40 ± 11 times higher than the supported one (standard deviation). Given its origin, ^210^Pb is mainly found in superficial environments. Indeed also in moraine sediments, exposed to the atmosphere, an unsupported contribute is present, but in this case the ratio between the two fractions is 3.3 (Fig. 2). If the only scavenging effect of precipitation explains the presence of unsupported ^210^Pb in the moraine sediments, this is not the case for cryoconite, where the accumulation of unsupported ^210^Pb is too high. High activities of unsupported ^210^Pb are typical in matrices whose composition is strongly influenced by deposition from the atmosphere, as lichen, moss or peat, but even in these cases they don’t exceed few hundred^22^ *Bq kg* ^−1^, an order of magnitude lower with respect to what observed in cryoconite. ^232^Th decay chain, where no long-lived mobile radionuclides are encountered, reveals a substantial equilibrium (Figs 2 and 3).

Also artificial radionuclides were detected: ^137^Cs, ^241^Am, ^207^Bi (see Figs 2a and 4). ^137^Cs is globally found in the environment as a product of atmospheric nuclear tests and nuclear accidents, it is a high-yield fission product from ^235^U. Its chemical properties (solubility and volatility) and relatively long half-life (30.1 yr) made this radionuclide extremely mobile and able to disperse worldwide^24, 25^. In Europe a relevant role in determining its diffusion was played by the Chernobyl accident in 1986^26^.

The observation of ^241^Am and ^207^Bi is by far rarer. ^241^Am (T_1/2_ 432 yr) is produced through multiple neutron capture and decay from ^238^U. It was released in the environment through nuclear accidents, authorized discharges and nuclear weapon tests^27^. As a consequence of the concurrent decay of its parent nuclide (^241^Pu, T_1/2_ 14.3 yr), its activity in the environment is not decreasing, but it is expected to peak by the end of 21^st^ century^28^. The occurrence of ^207^Bi (T_1/2_ 31.6 yr) is even more rare and poorly investigated. No more than 30 papers report about its occurrence^29, 30^. Its nucleosynthesis pathway is debated, but many evidences point to a role played by specific thermonuclear tests^31^, as the explosion of the Tzar thermonuclear device in 1961, occurred in Novaja Zemlya. This event is the prime suspected to explain the presence of ^207^Bi in Europe^32^.

Average (and standard deviation) activities of ^137^Cs, ^241^Am and ^207^Bi in cryoconite found on the Morteratsch glacier are respectively: 2.7(3.8) *kBq kg* ^−1^, 30(35) *Bq kg* ^−1^, 12(6) *Bq kg* ^−1^. The high deviations reflect the significative differences found among the samples. As in the case of ^210^Pb, also for these artificial nuclides the observed activities are remarkable. One sample in particular (CR3) showed maximum activities for all the 3 considered nuclides: 13.6 ± 0.7 *kBq* *kg* ^−1^ for ^137^Cs, 120 ± 9 *Bq kg* ^−1^ for ^241^Am and 25 ± 5 *Bq kg* ^−1^ for ^207^Bi. These values are among the highest ever observed in the environment; the highest one in the case of Am (with the exclusion of nuclear test and accident sites). Values reported in literature usually are in the range of 0.5–600 *Bq* *kg* ^−1^ for ^137^Cs^33, 34^, 0.05–3 *Bq* *kg* ^−1^ for ^241^Am^35^, 0.2–5 for ^207^Bi^29, 32^ (all data corrected for January 2017), one or two orders of magnitude lower then cryoconite.

As shown in Fig. 2a, activities of cryoconite are always well above what observed in moraine sediments, where it was not possible to detect ^207^Bi. The enrichment is particularly high for ^137^Cs. In sample CR3 ^137^Cs concentration is more than 2500 times higher with respect to the moraine material, while on average the ratio between activities of cryoconite and moraine sediments is 250, with a strong variability (standard deviation 720). Considering environmental samples, only other data relative to cryoconite collected on Austrian (Oher Dachstein massif^16^) and Italian (Val d’Aosta region^36^) glaciers are comparable to our results. In Fig. 4 it can be seen that the activities of the three radionuclides are strictly related to each other. The coefficients of determination (R^2^) of linear regression calculated for each pair of artificial nuclides ranges from 0.78 for Am-Bi, to 0.94 for Cs-Am. What distinguishes the different Alpine contexts is the slope of the regression curve. While Italian and Swiss samples share similar coefficients, close to global fallout reference^29, 37^, Austrian samples present higher relative ^137^Cs activities. This is probably related to the differential dispersion of radio-cesium after the Chernobyl accident. Due to geographical and meteorological issues Austrian Alps were more affected by Cs deposition with respect to Western and Central ones^38^. Taking into account the nuclides related to global fallout (the pair Am-Bi, Fig. 4c), a substantial homogeneity is observed. This reveals that cryoconite is capable to record events associated both to regional and global atmospheric phenomena.

### Elemental composition

An overview of the results concerning elemental composition is given in Fig. 2b. Elements were divided as follows (the element order of each group depends on the average concentration found in the samples): crustal elements, sub-divided into major (Si, Fe, Ca, Na, K) and trace ones (V, Rb, La, Ce, Sc, Cs); heavy elements (Hf, Th, U W, Ta and Au); anthropic ones (Zn, As, Se, Sb, Hg). Crustal elements are mostly associated to Earth crust, their biogeochemical cycle is only marginally affected by human activities. The heavy element group is quite heterogeneous. Hf, Th, U and Ta are considered geochemically incompatible^39^, their origin is mainly crustal and they are associated to heavy and resistant minerals. W, as also Ta, is a refractory element, characterized by extremely high melting point and resistance. Its behavior in the environment and the impact of human activities on its geochemistry are not yet well constrained, as in the case of gold. The last group, referred as “anthropic” elements, includes elements whose biogeochemical cycle is strongly influenced by human activities and anthropogenic emissions^40, 41^.

### Crustal elements

For most of these elements moraine sediments show higher concentration than cryoconite (Fig. 2b). This could be related to the presence of organic matter in cryoconite which dilutes the mineral fraction. Na and Ca present the higher degree of depletion, their concentration in cryoconite is halved with respect to moraine sediments. Being both quite soluble, such a significative depletion can be related to water running on glacier surface. The flowing of liquid water washes out soluble species from cryoconite, as Na and Ca. In accordance to their solubility, a depletion would be also expected for other alkali elements (as K, Rb and Cs), but this is not the case, on the contrary they are enriched in cryoconite. The process responsible for accumulation of radioactive cesium probably accounts also for the concentration of chemically similar species, as natural Cs and other alkali elements. Comparing the composition of cryoconite and moraine sediments it appears that the affinity degree for clay is an important factor in governing the accumulation of ionic soluble species in cryoconite (see Fig. 5a). Elements with high affinity for clay (Cs and Rb^42, 43^) are adsorbed from meltwater and concentrated in cryoconite. On the opposite, solubilization prevails on absorption for those elements with lower affinity, determining a depletion.

### Heavy elements

They are more abundant in cryoconite than in the moraine sediments (respectively +5, +108, +96, +30, +33 and +550% for Hf, Th, U, W, Ta, Au). This is an additional evidence about the effect of water flowing on the surface of the glacier during summer. Meltwater does not only remove soluble species from cryoconite, but also the lighter mineral fractions, which are easily mobilized. The removal of lighter minerals determines a relative enrichment of heavy minerals, where many trace elements are found at high concentration^44^. A similar process was noted to occur also in the atmosphere, where elements as Ta, Zr and Hf (associated to heavy minerals) are naturally found in excess in background aerosol (see Vlastelic *et al*.^45^ and references therein). Through different processes (chemical and physical) liquid water removes soluble, mobile and light elements both in the atmosphere and on the ground. A hypothetical influence of anthropic activities has to be ruled out, since the human impact on the mobilization of such elements is not relevant^40^. The only exception concerns gold. The environmental behavior of this noble and rare metal is definitely poorly investigated, despite it is estimated that the anthropogenic contribution to its biogeochemical cycle represents more than 90% of total^40^. Gold concentration in cryoconite is on average 6.5 times higher than in moraine sediments and 8.5 times higher than average upper continental concentration (UCC^46^), pointing to a non crustal contribution.

### Anthropic elements

Similarly to what observed for gold, also the elements grouped as anthropic show anomalous concentration in cryoconite. As shown in Fig. 5b, absolute concentrations and crustal enrichment factors highlight that the presence of these elements in cryoconite cannot be solely attributed to its mineral fraction. On the opposite in the moraine sediments they present a concentration perfectly comparable to UCC. The presence of these elements in cryoconite cannot be explained neither in the light of bare atmospheric deposition. Moraine sediments, being continuously exposed to the atmosphere, can be intended as a monitor for local atmospheric deposition. It doesn’t present any significant enrichment, as also noted for radionuclides. The elements found in excess in cryoconite (Zn, As, Sb, Se and Hg) are quite mobile and volatile and are largely emitted in the atmosphere as a consequence of mining, metal production, fossil fuel combustion and other activities^41^. Volatility and mobility are indeed important features in determining the atmospheric behavior of an element. Not by chance Zn, As, Sb, Se and Hg, among the elements whose biogeochemical cycle is more affected by anthropogenic disturbances^40^, share relatively low melting point and high volatility. Several observations demonstrated that Alpine ice and snow are effectively contaminated with these and other chemically related metals and metalloids, as Zn, Cd, Bi, Pb^12^. But concentrations found in cryoconite are 5 orders of magnitude higher than the ones observed in ice and snow.

### Carbonaceous Content

Data about organic and elemental carbon (OC and EC) in cryoconite are found in Fig. 2c. Organic carbon content ranges from 3.5 to 5.7% m/m. From OC, total OM can be inferred, assuming that 50% of organic matter is composed by organic carbon^47^. Average OM in cryoconite samples is 9.5% of dry mass (first datum ever relatively to the Alps), with a standard deviation of 1.6%, in perfect agreement with other continental glaciers^3^. Concerning elemental carbon no previous data were available for cryoconite. Elemental carbon, strictly related to black carbon^48^, is of extreme importance in relation to the optical properties of glaciers, since it strongly adsorbs visible light, altering the radiative budget of ice and snow surfaces where it is found^49^. Average concentration of EC is 0.49 (0.25) % of dry mass (standard deviation). Two samples, CR10 and CR11, present peculiar features, with low OC content (below 4%) and relatively higher concentration of EC (close to 1%). The low content of OC may be related to a recent formation of these cryoconite holes, where the microbiological community is still at its early stages. Elemental and black carbon were already found in ice and snow samples from the Alps, as a consequence of combustion processes and atmospheric transport to high altitude regions^50^, but typical concentrations were in the range of 10^0^–10^2^ ng g^−1^, 8–9 orders of magnitude lower than what observed in cryoconite^51^. Finding a so relevant amount of EC supports the hypotheses proposed by Cook *et al*.^3^ about the entrainment of EC in cryoconite, but it also suggests that it could represent an important source of carbon for cryoconite microbial communities. In the Morteratsch pro-glacial plain, BC represents an essential source of carbon for the developing soils^52^. The origin of such a substantial amount was not constrained, but cryoconite could be involved. As long as it remains on the glacier, cryoconite accumulates OC and EC, but when, due to glacier retreat, it is finally released in the environment, it represents an important source of carbon for the pro-glacial environment, where few other sources are available.

### Multivariate analysis

To better comprehend the relationships between the different analyzed species, principal component analysis (PCA) was applied. Results concerning the first two components are presented in Fig. 6. The first one (PC1, explained variance 32%) is mainly related to natural radioactivity (K and Th-U decay chains, including ^210^Pb) and secondarily to OC. Interestingly ^210^Pb and OC seem tightly correlated (Pearson correlation coefficient, r = 0.79), confirming that organic matter plays a relevant role in adsorbing lead^53^. PC1 efficiently separates moraine sediments from cryoconite samples, but it only distinguishes them as a whole, no further details are noticed. Conversely the second component (PC2, explained variance 26%) presents a finer resolving power (Fig. 6). Indeed not only moraine sediments are at one of the two extremes of the sample distribution, but cryoconite samples are also displayed along a preferential direction. The original variables with the highest loadings in PC2 are artificial radionuclides, anthropic elements, Au and EC, i.e. all the species whose main origin is associated to anthropic activities and which are enriched in cryoconite. Such a picture suggests that this component can be interpreted as a “pollution” index.

### On the accumulation capability of cryoconite

Our results show that cryoconite can accumulate radioactive isotopes, specific elements and elemental carbon with unprecedented efficiency. The comparison between cryoconite and moraine sediments ruled out the possibility to associate such strong enrichments to anomalous atmospheric depositional fluxes or to composition anomalies related to the local mineral material. Atmospheric deposition certainly plays an important role in influencing the composition of cryoconite, since species as unsupported ^210^Pb, whose origin is essentially atmospheric^22^, are found in abundance (Figs 2 and 3). But from previous observations it is deduced that atmospheric deposition alone is not sufficient to explain such an accumulation. Alternative and complementary processes need to be taken into account. The only reasonable source of impurities other than direct atmospheric deposition, is meltwater. A glacier can be seen as a temporary repository of atmospheric deposited matter, having snow, ice and their impurities (including pollutants), a substantial atmospheric origin. Ablation mobilizes and releases into the environment what is preserved in the glacier through meltwater. Taking ^210^Pb as an example, it can be assumed that the local depositional flux ranges from 50 to 150 *Bq* *m* ^−2^ *yr* ^−122^, with an annual deposition on the glacier of 1–2 *GBq* (surface of Morteratsch glacier from Zekollari *et al*.^54^). During the melting season a fraction of this amount, including the deposition from previous years, is mobilized and transported downward along the surface of the glacier, where cryoconite is found. The latter is the only available substrate on the glacier which natural and anthropic substances can be adsorbed on. It can be hypothesized that a part of transported ^210^Pb is retained in cryoconite, determining the enrichments we observed. This conceptual scheme not only fit for lead, but for all the impurities transported with meltwater and presenting affinity for cryoconite.

The differential enrichment of cationic species (Fig. 5a) and the relevant accumulation of natural and radioactive Cs, the element with the highest affinity for clay^55^, are indicative of the important role played by clay minerals, a major constituent of cryoconite^56, 57^. Conversely the high enrichments of elements as Pb, Sb, Se and Hg, can be related to the organic component which is present in cryoconite, since all of them present affinity for it^53, 58–60^. PCA (Fig. 6) and Pearson coefficients (see SI) partly confirmed this scenario since OC and elements as Hg, As and ^210^Pb are positively correlated to it (r = 0.44; 0.82; 0.79 respectively). The correlation with other anthropic elements is lower (r = 0.33; 0.01; 0.36 for Zn, Se, Sb), but the variability of OC content in cryoconite samples is low (see Fig. 3c). What is important is the presence of OM, not its quantity, which is a quite stable parameter.

The contemporary presence of fractions with specific affinities for different substances and the seasonal availability of large quantities of atmospheric related impurities are the main factors which account for the high accumulation capability of cryoconite. Such features make it a temporary sink for many inorganic and organic substances stored in glaciers. They could be used for long-term atmospheric monitoring purposes, also in relation to exotic species, since they proved to be able to preserve extremely rare radionuclides as ^207^Bi, released in the environment only punctually^29, 30^. Models attempting to reproduce the secondary mobilization of pollutants into the environment^61^, should take into account cryoconite, which, in the light of our result, delay and concentrate the final release from the glacier.

### Heterogeneity, a question of age?

What remains unclear is the high variability observed among different cryoconite holes. In Figs 4 and 5 it can be seen that the concentration of artificial radionuclides and anthropic elements is quite heterogeneous. Looking at the data it can also be appreciated that despite being extremely variable, a close correlation exists between different species. A striking case concerns artificial radionuclides (see Fig. 4). PCA shows that most of the anthropic species are positively correlated among each other. Looking at the samples (Fig. 6b), PC2, interpreted as a “pollution index”, is capable to separate them in function of their pollution content. Moraine sediments present the lowest value, while sample CR3 (where maximum activities of artificial radionuclides were found) the highest one. The only reasonable factor which could explain the different degree of contamination is the age of cryoconite. Considering the selection of sampling sites, the influence of surficial hydrology should not be relevant. Also the OM content doesn’t seem important, since its variability is low (Fig. 2c). The older is the cryoconite hole and the higher is the accumulation of pollutants. Up to now no successful attempts to determine the formation age of cryoconite are known. Probably a maximum possible age, depending on several factors, exists in relation to each glacier^11^. On the Alps this age cannot exceed few decades. This limit is posed by glacier dynamics, since the movement of ice and its melting prevent the preservation of older glacier surfaces where cryoconite can accumulate. Few years, at most few decades, could be sufficient to induce a significative heterogeneity, as observed on the Morteratsch glacier.

Determining the age of cryoconite remains a challenging target. It could shed important lights on the dynamics which involve ablation, the release of legacy pollutants and their accumulation in cryoconite, but also on the cycling of relevant atmospheric species and on their environmental fate, without forgetting the important role of microbiological communities.

## Methods

### Samples and location

Cryoconite samples were collected during summer 2015 and 2016 from the Vadret da Morteratsch, a north faced glacier located in the Bernina Massif (Swiss Alps). Cryoconite was sampled from the terminal part of the glacier, located between 2100 and 2400 m.a.s.l. and subjected to intense seasonal melting (Fig. 1). Each sample represents a distinct cryoconite hole. Samples were gathered from the central region of the ice body, where the surface of the glacier presented the simplest possible hydrological features. Areas close to moulins and main bedières were avoided. An additional sample of fine glacial diamicton was collected from a lateral moraine lying few meters from the glacier. Samples were kept frozen until they were processed for successive measurements. After melting, samples were dried at 70° for 12 hours and successively sieved to remove the coarser fraction (>1 mm). Three aliquots were obtained: one for γ-spectroscopy (≈1 g), one for Instrumental Neutron Activation Analysis (INAA, ≈0.2 g), one for carbon analysis (few grams).

### Gamma-spectroscopy and radioactivity

Gamma-radioactivity of cryoconite samples was measured with a high purity germanium (HpGe) well detector. The instrument is dedicated to low background measurements^62–64^. A full description of the instrument and of its customization to reduce the radioactive background is presented here^64^. Given the low amount of available material and the low concentration of some radionuclides, the application of low background techniques was mandatory not only because of the low amount of available material, but also to achieve an appropriate signal to background ratio in a reasonable time^64^. Samples were counted for 5–7 days. Before counting, they were sealed in closed vials so as to allow the secular equilibrium between ^222^Rn and its progenies to be reached^23^. To determine radioactive activity, absolute efficiencies were calculated at each γ-line of interest through Monte Carlo simulations^63^, using a GEANT4 based code^65^, which reproduce the instrumental apparatus, a virtual sample, the decay of the selected radionuclides and the radiation detection process by the crystal. According to this protocol differential self-adsorption phenomena and coincidence summing events are intrinsically taken into account. Further details about the procedure and the processing of γ-spectra are found here^63, 64^, including information about calibration and quality assurance. Emissions of interest were selected so as to minimize interferences. Only for ^224^Ra (241.0 keV) it was necessary to apply a correction to eliminate an interference from ^214^Pb. All data are corrected for decay to January 2017; full details are found in the Supporting Information.

### Neutron Activation

The elemental composition was determined through Instrumental Neutron Activation Analysis (INAA). Neutron irradiation was performed at LENA laboratories^66^ (University of Pavia). To detect both short-lived and long-lived radionuclides two irradiations were planned, following the scheme adopted by Baccolo *et al*.^64^. The acquisition of γ spectra was carried out at different times both at LENA and at the Radioactivity laboratory of Milano-Bicocca university. Calculation of elemental concentrations was done in accordance to a relative method, as fully described here^67^. Twenty elements were quantified, details are presented in the SI. All nuclear data necessary to analyze and process the results presented in this work were taken from Chu *et al*.^68^.

### Organic and elemental carbon

For Elemental Carbon (EC) and Organic Carbon (OC) determination, a Thermo-Optical Sunset EC/OC analyzer (Sunset Lab Inc.) was used, following the NIOSH 5040 protocol^69^. Cryoconite powder samples were suspended on quartz fiber filters (Pall, 2500QAO-UP, 47 mm diameter) pre-fired at 700 °C for 1 h in order to remove any possible OC contamination^70^. All the filters were weighed before and after cryoconite deposition in an air-conditioned room (T = 20 ± 1 °C; rel. um. = 50 ± 5%), after 48 h conditioning. The gravimetric determination of cryoconite mass was performed using an analytical microbalance (precision: 1 μg) operated inside the conditioned room; electrostatic effects were avoided by the use of a deionizing gun. Combining the information about the mass of cryoconite deposited on the filters and about the EC/OC surface concentration, m/m concentration was calculated.

## Electronic supplementary material

Supplementary Material

Supplementary Database
