# Author Correction: Cryoconite as a temporary sink for anthropogenic species stored in glaciers

**DOI:** 10.1038/s41598-018-24613-7

**Published:** 2018-04-25

**Authors:** Giovanni Baccolo, Biagio Di Mauro, Dario Massabò, Massimiliano Clemenza, Massimiliano Nastasi, Barbara Delmonte, Michele Prata, Paolo Prati, Ezio Previtali, Valter Maggi

**Affiliations:** 1 Department of Environmental Sciences, University of Milano-Bicocca, P.zza della Scienza 1, 20126 Milano, Italy; 2 INFN, section of Milano-Bicocca, P.zza della Scienza 3, 20126 Milano, Italy; 3 Department of Physics, University of Genova, Via Dodecaneso 33, 16146 Genova, Italy; 4 INFN, section of Genova, Via Dodecaneso 33, 16146 Genova, Italy; 5 University of Milano-Bicocca, Physics Department, P.zza della Scienza 3, 20126 Milano, Italy; 6 LENA, University of Pavia, Via G. Aselli 41, 27100 Pavia, Italy

Correction to: *Scientific Reports* 10.1038/s41598-017-10220-5, published online 29 August 2017

In Figure 2c, the columns for CR3 and CR4 have been obscured. The correct Figure 2 appears below as Figure 1.

**Figure 1:**
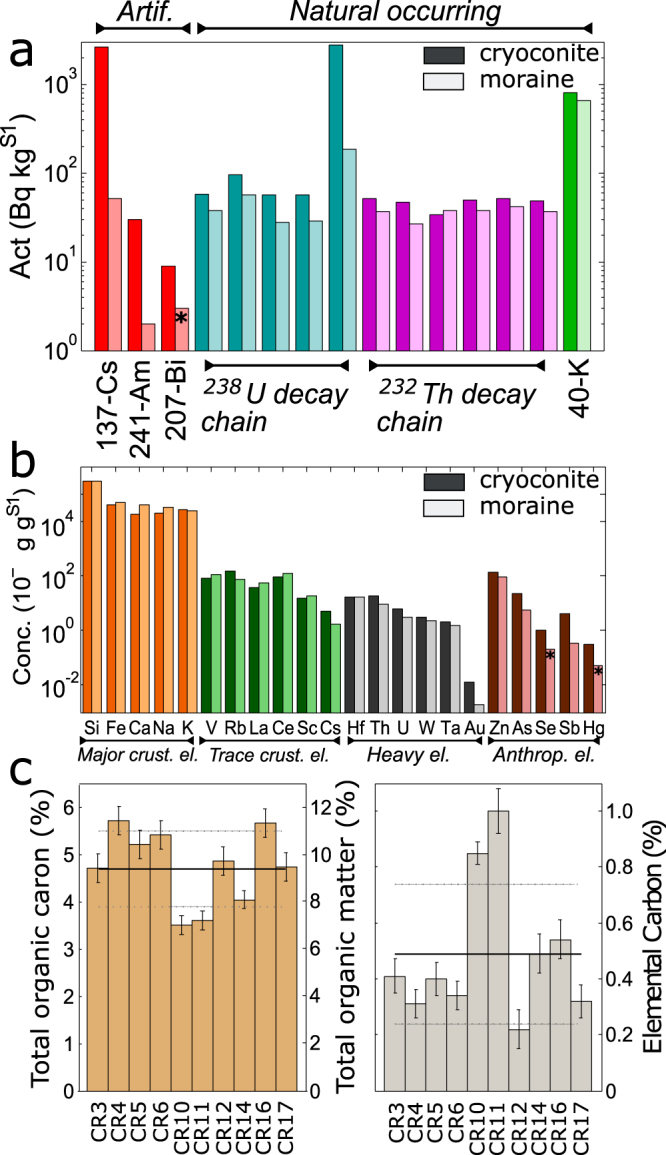
Average data about natural radioactivity (panel a), elemental composition (panel b) and carbonaceous content (panel c). In the first two panels average results about cryoconite and moraine sediments are presented (dark and light colors respectively). Not all nuclide labels are reported for U and Th chains, see Fig. 3 for the complete list. In panel c data related to single sample are shown, lines refer to average and standard deviation values. Asterisks highlight to values below detection limit.

