# Supplementary material for: Cryoconite as a temporary sink for anthropogenic species stored in glaciers
Source: Sci Rep. 2017 Aug 29;7:9623. doi: 10.1038/s41598-017-10220-5 (PMC5575069; doi:10.1038/s41598-017-10220-5)
Supplement: Supplementary file 1 — Supplementary Material [file 41598_2017_10220_MOESM1_ESM.pdf]

Supplementary Material to "Cryoconite as a temporary sink for anthropogenic species stored in glaciers"

Giovanni Baccolo[1,2*], Biagio Di Mauro[1], Dario Massabò[3,4], Massimiliano Clemenza[3,5], Massimiliano Nastasi[3,5], Barbara Delmonte[2], Michele Prata[6], Paolo Prati[3,4], Ezio Previtali[3,5], Valter Maggi[1,2]

1. University of Milano-Bicocca, Department of Environmental Sciences, P.zza della Scienza 1, 20126, Milano, Italy
2. INFN, section of Milano-Bicocca, P.zza della Scienza 3, 20126, Milano, Italy
3. Department of Physics, University of Genoa, Via Dodecaneso 33, 16146 Genoa, Italy
4. INFN, section of Genova, Via Dodecaneso 33, 16146 Genoa, Italy
5. University of Milano-Bicocca, Physics Department, P.zza della Scienza 3, 20126, Milano, Italy
6. LENA, University of Pavia, Via G. Aselli 41, 27100, Pavia, Italy

* corresponding author, email: giovanni.baccolo@mib.infn.it

Tab. S1 Main information about the detection of γ-radioactivity in cryoconite samples. For each radionuclide the associated decay chain, its half life, efficiency calculated through Monte Carlo simulations (corresponding to absolute efficiency multiplied by the probability of emission of the considered γ-line, i.e. the branching ratio, B.R.[1]), detection limits and average uncertainties are reported. Detection limits were calculated in accordance to Currie[2]. Nuclear data were taken from here[3].

Decay Chain	Radionucl.	$T_{1/2}$	γ-line energy (keV)	Eff. · B.R. (%)	Detection Limit (Bq Kg^{-1})	Average Uncertainty (%)
^{238}U – natural	^{210}Pb	22.3 yr	46.5	2.8	17.3	5.9
none – artificial	^{241}Am	432.2 yr	59.5	28.2	1.7	13
^{238}U – natural	^{234}Th	24.1 d	92.3-92.8	2.8	17.7	13
^{232}Th – natural	^{212}Pb	10.64 hr	238.6	24.6	1.9	7.8
^{232}Th – natural	^{224}Ra	3.66	241.0	25.8	21.1	35
^{238}U – natural	^{214}Pb	26.8 min	295.2	9.5	4.7	12
^{232}Th – natural	^{228}Ac	6.15 hr	338.3	4.2	11.5	17
^{238}U – natural	^{214}Pb	26.8 min	351.9	15.0	3.1	9.1
^{232}Th – natural	^{208}Tl	3.05 min	583.2	3.2	7.4	16
^{238}U – natural	^{214}Bi	19.9 min	609.3	5.2	4.6	13
none – artificial	^{137}Cs	30.07 yr	661.7	20.4	0.7	5.5
^{232}Th – natural	^{212}Bi	60.55 min	727.3	1.1	11.2	27
^{232}Th – natural	^{208}Tl	3.05 min	860.6	0.6	17.1	39
^{232}Th – natural	^{228}Ac	6.15 hr	911.2	3.7	2.9	13
none – artificial	^{207}Bi	31.55 yr	1063.7	4.5	2.6	32
none – natural	^{40}K	$1.3 \cdot 10^9$ yr	1460.8	1.3	6.6	7.4
^{238}U – natural	^{214}Bi	19.9 min	1764.5	1.6	4.4	15
^{232}Th – natural	^{208}Tl	3.05 min	2614.5	0.7	7.9	28

Tab. S2 Radioactivity determined in cryoconite samples. All data are expressed in *Bq kg^{-1}*. Uncertainties are reported in bracket. Detection limits are reported when activity was below the analytical sensitivity. In the case of ^{238}U and ^{232}Th (marked by an asterisk) activities were not determined through γ-spectrometry, they were calculated converting total U and Th elemental concentrations (determined through INAA) into radioactivity, using the fundamental law of radioactive decay. Such data are missing for samples CR13 and CR15 since for these samples only environmental radioactivity was measured, not their elemental composition. All data corrected for January 2017.

	**CR3**	**CR4**	**CR5**	**CR6**	**CR10**	**CR11**	**CR12**	**CR13**	**CR14**	**CR15**	**CR16**	**CR17**	**MS1**
**^{137}Cs**	13600(700)	480(30)	480(30)	510(30)	3500(200)	5300(300)	325(20)	400(20)	2050(110)	3400(200)	1010(50)	685(40)	51(4)
**^{241}Am**	120(9)	5.7(1.0)	2.9(0.6)	5.4(0.9)	44(3)	65(5)	5.1(0.7)	3.1(0.7)	27(2)	59(5)	15(2)	10(1)	*<2*
**^{207}Bi**	25(5)	9(4)	*<3*	11(5)	14(3)	16(4)	*<3*	8(3)	8(2)	9(3)	6(2)	*<3*	*<3*
**^{40}K**	720(60)	770(60)	750(60)	750(60)	850(60)	790(60)	870(60)	900(60)	790(50)	870(70)	800(60)	820(60)	660(50)
**^{238}U***	60(5)	61(5)	53(5)	59(5)	77(6)	118(9)	49(4)	-	57(4)	-	82(6)	38(2)	38(2)
**^{234}Th**	85(20)	113(15)	95(11)	101(12)	100(14)	97(13)	77(11)	85(14)	92(10)	76(13)	88(14)	99(13)	57(7)
**^{214}Pb**	43(4)	57(5)	55(5)	60(5)	62(5)	53(5)	68(5)	65(5)	60(4)	53(5)	51(4)	62(5)	28(3)
**^{214}Bi**	42(5)	57(6)	58(6)	59(6)	60(5)	52(6)	68(6)	67(6)	59(5)	57(7)	45(5)	63(6)	29(3)
**^{210}Pb**	2400(150)	3400(200)	3600(200)	3400(200)	2120(120)	2380(150)	1650(100)	2100(130)	2900(150)	2220(140)	2800(160)	4200(240)	165(20)
**^{232}Th***	54(4)	59(4)	46(4)	50(4)	63(2)	101(5)	69(3)	-	66(2)	-	69(3)	71(3)	37(3)
**^{228}Ac**	56(6)	41(6)	45(6)	42(6)	47(5)	41(5)	44(4)	51(5)	47(4)	50(6)	44(4)	51(5)	27(8)
**^{224}Ra**	50(25)	37(15)	36(15)	26(15)	35(13)	*<21*	21(11)	28(12)	32(11)	58(22)	*<21*	42(13)	38(10)
**^{212}Pb**	53(5)	48(4)	49(4)	51(4)	49(4)	45(4)	50(4)	55(4)	52(4)	50(4)	47(4)	55(4)	38(3)
**^{212}Bi**	65(20)	44(15)	41(13)	53(15)	49(11)	39(12)	55(12)	49(13)	51(10)	50(15)	63(15)	63(14)	42(7)
**^{208}Tl**	43(6)	55(8)	44(7)	53(8)	51(5)	47(6)	45(5)	52(6)	49(6)	53(8)	44(6)	55(6)	37(5)

Tab. S3 Nuclear reactions used to quantify the elemental concentrations within the samples. Half-lives and γ-energies from here[3].

Isotope	Reaction	$T_{1/2}$	Gamma Emission (keV)
^{23}Na	$^{23}Na(n,\gamma)^{24}Na$	15.0 h	1368.6-2754.0
^{29}Si	$^{29}Si(n,p)^{29}Al$	6.6 min	1273.4
^{41}K	$^{41}K(n,\gamma)^{42}K$	12.4 h	1524.7
^{45}Sc	$^{45}Sc(n,\gamma)^{46}Sc$	83.8 d	889.3-1120.5
^{46}Ca	$^{46}Ca \rightarrow ^{47}Sc^{a}$	3.3 d	159.4
^{51}V	$^{51}V(n,\gamma)^{52}V$	3.7 min	1434.1
^{54}Fe	$^{54}Fe(n,p)^{54}Mn$	312.3 d	834.8
^{58}Fe	$^{58}Fe(n,\gamma)^{59}Fe$	44.5 d	1099.2-1291.6
^{64}Zn	$^{64}Zn(n,\gamma)^{65}Zn$	244.3 d	1115.5
^{74}Se	$^{74}Se(n,\gamma)^{75}Se$	119.8 d	400.7
^{75}As	$^{75}As(n,\gamma)^{76}As$	1.08 d	559.1
^{85}Rb	$^{85}Rb(n,\gamma)^{86}Rb$	18.6 d	1076.6
^{121}Sb	$^{121}Sb(n,\gamma)^{122}Sb$	2.7 d	564.1
^{123}Sb	$^{123}Sb(n,\gamma)^{124}Sb$	60.2 d	602.7-1691.0
^{133}Cs	$^{133}Cs(n,\gamma)^{134}Cs$	2.1 y	604.7-795.9
^{139}La	$^{139}La(n,\gamma)^{140}La$	1.7 d	1596.2
^{140}Ce	$^{140}Ce(n,\gamma)^{141}Ce$	32.5 d	145.4
^{180}Hf	$^{180}Hf(n,\gamma)^{181}Hf$	42.4 d	345.9-482.2
^{181}Ta	$^{181}Ta(n,\gamma)^{182}Ta$	114.4 d	1189.1
^{186}W	$^{186}W(n, \gamma)^{187}W$	23.7 h	685.8
^{197}Au	$^{197}Au(n, \gamma)^{198}Au$	2.7 d	411.8
^{202}Hg	$^{202}Hg(n,\gamma)^{203}Hg$	46.6 d	279.2
^{232}Th	$^{232}Th \rightarrow ^{233}Pa^{b}$	27.0 d	300.3-312.2
^{238}U	$^{238}U \rightarrow ^{239}Np^{c}$	2.4 d	106.1-228.2-277.6

[a] Entire reaction $^{46}Ca(n,\gamma)^{47}Ca \xrightarrow{\beta^-} {}^{47}Sc$

[b] Entire Reaction $^{232}Th(n,\gamma)^{233}Th \xrightarrow{\beta^-} {}^{233}Pa$

[c] Entire Reaction $^{238}U(n,\gamma)^{239}U \xrightarrow{\beta^-} {}^{239}Np$

Tab. S4 Composition of the samples determined through INAA. Uncertainties are reported in brackets. Please note that the unit is not the same for all the elements. When it was not possible to determine the elemental content the upper limit of concentration is reported. CR samples are cryoconite ones, MS1 is the sample collected from the moraine.

Element	CR3	CR4	CR5	CR6	CR10	CR11	CR12	CR14	CR16	CR17	MS1
**Na** (mg/g)	14.2(0.8)	18(1)	16.3(0.9)	18(1)	15(1)	17(2)	13(1)	16(2)	17.0(1.5)	35(4)	31(2)
**Si** (mg/g)	275(75)	315(85)	360(95)	360(95)	320(65)	270(65)	275(55)	245(50)	215(40)	370(65)	315(90)
**K** (mg/g)	24(3)	24(3)	24(3)	25(4)	24(6)	27(6)	22(5)	28(6)	24(6)	30(7)	23(4)
**Ca** (mg/g)	19(1)	19(1)	21(1)	22(1)	19(4)	24(5)	10(2)	17(3)	26(5)	13(3)	40(2)
**Sc** (μg/g)	18(2)	15(1)	16(1)	16(1)	14.7(0.9)	18(1)	11.7(0.8)	14.1(0.9)	19(1)	10.3(0.6)	19(2)
**V** (μg/g)	125(15)	82(11)	108(14)	88(12)	88(14)	110(20)	52(8)	80(15)	85(15)	53(9)	100(15)
**Fe** (mg/g)	46(2)	40(2)	42(2)	41(2)	37(1)	45.8(1.4)	32.6(1.2)	40.8(1.2)	50.5(1.4)	27.7(0.9)	46(2)
**Zn** (μg/g)	275(20)	130(9)	160(11)	164(11)	140(10)	155(10)	98(10)	140(10)	166(11)	81(7)	87(6)
**As** (μg/g)	19(3)	25(3)	23(3)	25(3)	16(3)	17(3)	16(3)	24(4)	26(4)	31(5)	5.3(0.3)
**Se** (μg/g)	1.5(0.3)	0.7(0.2)	0.7(0.2)	0.4(0.1)	1.4(0.3)	1.6(0.4)	0.4(0.2)	1.0(0.2)	0.9(0.2)	0.6(0.2)	< 0.2
**Rb** (μg/g)	115(10)	127(11)	113(10)	141(12)	153(8)	146(8)	209(12)	170(9)	135(7)	104(6)	74(7)
**Sb** (μg/g)	9(1)	3.7(0.3)	3.6(0.5)	3.8(0.6)	5.8(0.3)	6.0(0.4)	2.2(0.1)	4.2(0.2)	4.4(0.3)	2.8(0.2)	0.2(0.1)
**Cs** (μg/g)	6.6(0.5)	4.9(0.4)	4.7(0.3)	4.4(0.3)	5.2(0.2)	5.7(0.3)	4.2(0.2)	5.3(0.2)	5.9(0.3)	3.9(0.2)	1.6(0.1)
**La** (μg/g)	44(2)	41(2)	35.1(0.8)	36(2)	34(4)	45(5)	17(2)	37(4)	37(4)	47(5)	57(3)
**Ce** (μg/g)	92(7)	92(7)	74(6)	80(6)	79(6)	103(8)	65(5)	87(6)	92(6)	104(8)	123(9)
**Hf** (μg/g)	11(1)	11(1)	10(1)	13(2)	16(2)	17(2)	20(2)	11(1)	18(2)	19(2)	16(2)
**Ta** (μg/g)	2.0(0.4)	2.1(0.4)	1.8(0.3)	1.8(0.3)	1.5(0.2)	1.5(0.2)	2.6(0.3)	1.9(0.2)	2.0(0.2)	2.0(0.2)	1.5(0.3)
**W** (μg/g)	6(1)	4(1)	14(3)	4.3(0.9)	2.9(0.6)	3.0(0.5)	2.4(0.4)	3.3(0.5)	3.7(0.6)	2.0(0.4)	2.2(0.6)
**Au** (ng/g)	28(5)	4.1(0.9)	14(3)	7(1)	21(6)	15(5)	5(3)	16(5)	10(3)	9(3)	2.0(0.5)
**Hg** (μg/g)	1.0(0.2)	0.6(0.1)	0.32(0.08)	0.6(0.1)	0.41(0.06)	0.3(0.1)	0.18(0.04)	0.41(0.07)	0.3(0.1)	0.36(0.6)	< 0.1
**Th** (μg/g)	13(1)	15(1)	11.4(0.9)	12(1)	15.4(0.6)	32.3(1.2)	17.1(0.7)	16.2(0.6)	17.1(0.6)	17.4(0.7)	9(1)
**U** (μg/g)	4.7(0.4)	4.9(0.4)	4.2(0.4)	4.7(0.4)	6.1(0.5)	9.4(0.7)	3.9(0.3)	4.6(0.3)	6.6(0.5)	6.2(0.5)	3.1(0.2)

Tab. S5 Organic and elemental carbon content of cryoconite samples. Uncertainties are reported in bracket. Total organic matter content was calculated from organic carbon content, assuming the conversion factor proposed in Pribyl[4]. Given its exclusively mineral composition it was not possible to determine the carbonaceous content in the moraine sediments (MS1).

Sample	Organic Carbon (% m/m)	Organic Matter (% m/m)	Elemental Carbon (% m/m)
**CR3**	4.7(0.3)	9.4(0.6)	0.41(0.06)
**CR4**	5.7(0.3)	11.4(0.6)	0.31(0.05)
**CR5**	5.2(0.3)	10.4(0.6)	0.40(0.06)
**CR6**	5.4(0.3)	10.8(0.6)	0.34(0.05)
**CR10**	3.5(0.2)	7.1(0.4)	0.85(0.04)
**CR11**	3.6(0.2)	7.2(0.4)	1.00(0.08)
**CR12**	4.9(0.3)	9.7(0.6)	0.22(0.07)
**CR14**	4.0(0.2)	8.1(0.4)	0.49(0.07)
**CR16**	5.7(0.3)	11.3(0.6)	0.54(0.07)
**CR17**	4.7(0.3)	9.4(0.6	0.32(0.06)
**MS1**	n.d.	-	n.d.

References

1 Clemenza, M., Fiorini, E., Previtali, E. & Sala, E. Measurement of airborne 131I, 134Cs and 137Cs due to the Fukushima reactor incident in Milan (Italy). *J. Environ. Radioactiv.* **114,** 113-118 (2012).

2 Currie, L. A. Limits for qualitative detection and quantitative determination - Application to radiochemistry. *Anal. Chem.* **40,** 586-593 (1968).

3 Chu, S. Y. F., Ekstrom, L. P. & Firestone, R. B. The Lund/LBNL Nuclear Data Search,» [Online] www.nucleardata.nuclear.lu.se/toi, (1999).

4 Pribyl, D. W. A critical review of the conventional SOC to SOM conversion factor. *Geoderma* **156,** 75-83 (2010).
